# Recurrent dermatofibrosarcoma protuberans: challenging a surgeon’s dexterity for the ‘tricky’ margins

**DOI:** 10.3332/ecancer.2018.858

**Published:** 2018-08-13

**Authors:** Deepak Kumar Diwakar, Nikita Wadhwani, Shivani Paruthi

**Affiliations:** Department of General Surgery, Vardhman Mahavir Medical College and Safdarjung Hospital, New Delhi 110 029, India

**Keywords:** dermatofibrosarcoma protuberans (DFSP), breast tumours, oncosurgery, recurrent skin tumours

## Abstract

Soft tissue tumours represent 0.2%–1% of all breast malignancies. [Al Tarakji M, Toro A, and Di Carlo I, *et al* (2015)** Unusual presentation of dermatofibrosarcoma protuberans in a male patient’s breast: a case report and review of the literature** *World J Surg Oncol*** 13** 158 https://doi.org/10.1186/s12957-015-0562-1]. Out of those, Dermatofibrosarcoma protuberans (DFSP) of the breast is extremely rare, especially in men with only six cases, including this case, reported so far. We report a case of recurrent DFSP in a 35-year-old male after a latency of 8 years in the region of previous surgical scar. It was managed by a wide local excision followed by reconstruction using latissimus dorsi flap. It is important to carefully manage recurrent cases because the post-operative margin status is an important determinant of recurrence, and therefore, requires vigilant resection of the tumour without causing extensive morbidity to the patient.

## Introduction

Dermatofibrosarcoma protuberans (DFSP) is a rare soft tissue tumour with an incidence rate of 4.2–4.5 cases per million persons per year and local recurrence rates up to 60% [2]. It grows slowly and presents usually as a nodular superficial lesion on the trunk or the extremities. Although these tumours are locally aggressive, with a high rate of recurrence following surgery, the prognosis is considered excellent when it is effectively treated [3]. In this case report, we discuss a case of recurrent DFSP of the breast after a latency of 8 years and its surgical management in detail.

## Case summary

A 35-year-old male from Central India presented to the Surgical Outpatient Department with a gradually increasing nodule of 5 cm in the right mammary region. It was not associated with any pain, ulceration or discharge. He had a similar complaint in the same location 8 years ago and was diagnosed with biopsy-proven DFSP and was surgically treated for the same. He did not have the records of his previous surgery, but he had the histopathology report, which stated the diagnosis of DFSP with histologically negative margin status. According to the history given by the patient, he did well after the surgery and was disease free until he noticed this nodule few months ago. There was no history of radiotherapy and/or chemotherapy. He did not have any other co-morbidities. There was no family history of breast, colon or skin cancer.

**Figure 1.**
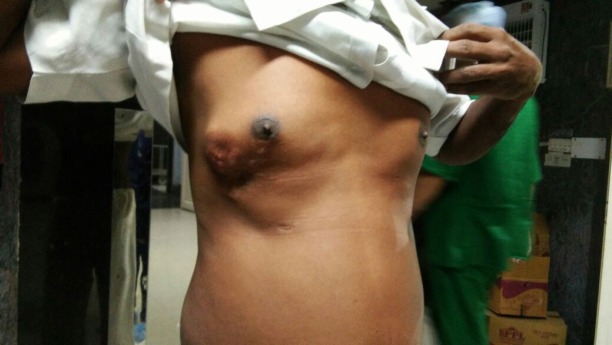
Pre-operative appearance—4 × 5-cm firm reddish-pink nodule, fixed with the overlying skin and mobile in all directions, 2-cm lateral to the right nipple-areola complex extending up to the anterior axillary lin.

He had a normal body mass index and his vital parameters and systemic examination was within normal limits. On examination, a 4 × 5-cm firm reddish-pink nodule, fixed with the overlying skin and restricted mobility, was present 2-cm lateral to the right nipple-areola complex extending up to the anterior axillary line (Figure 1). Since it was present over his previous scar, the anatomy of the scar was obscured. There was no axillary lymphadenopathy. Left breast was normal.

His complete blood count, liver function test and kidney function test were within normal limits. A chest radiograph and contrast-enhanced computed tomography (CT) scan were performed to evaluate local extension and metastasis, which were absent. Fine-needle aspiration cytology of the nodule revealed spindle cells. After a careful history, examination and diagnostic workup, a preoperative diagnosis of recurrent DFSP was made. Keeping the aggressive nature of the recurrent tumour and future reconstruction in mind, a wide local excision (WLE) with pedicled latissimus dorsi (LD) flap was planned, after consultation with the Plastic Surgery Department.

**Figure 2.**
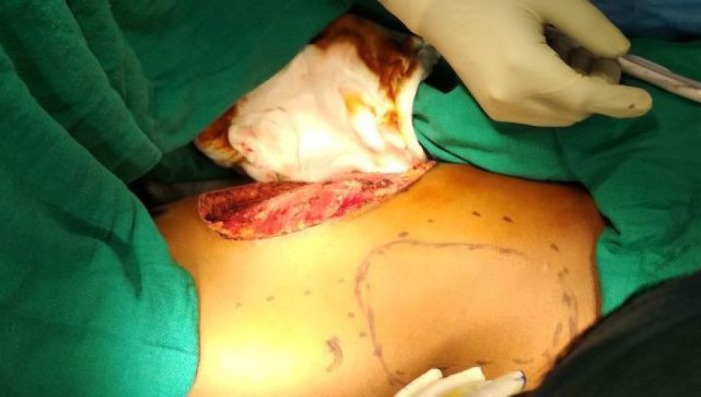
Intra-operative view of defect after WLE and marking for LD flap.

**Figure 3.**
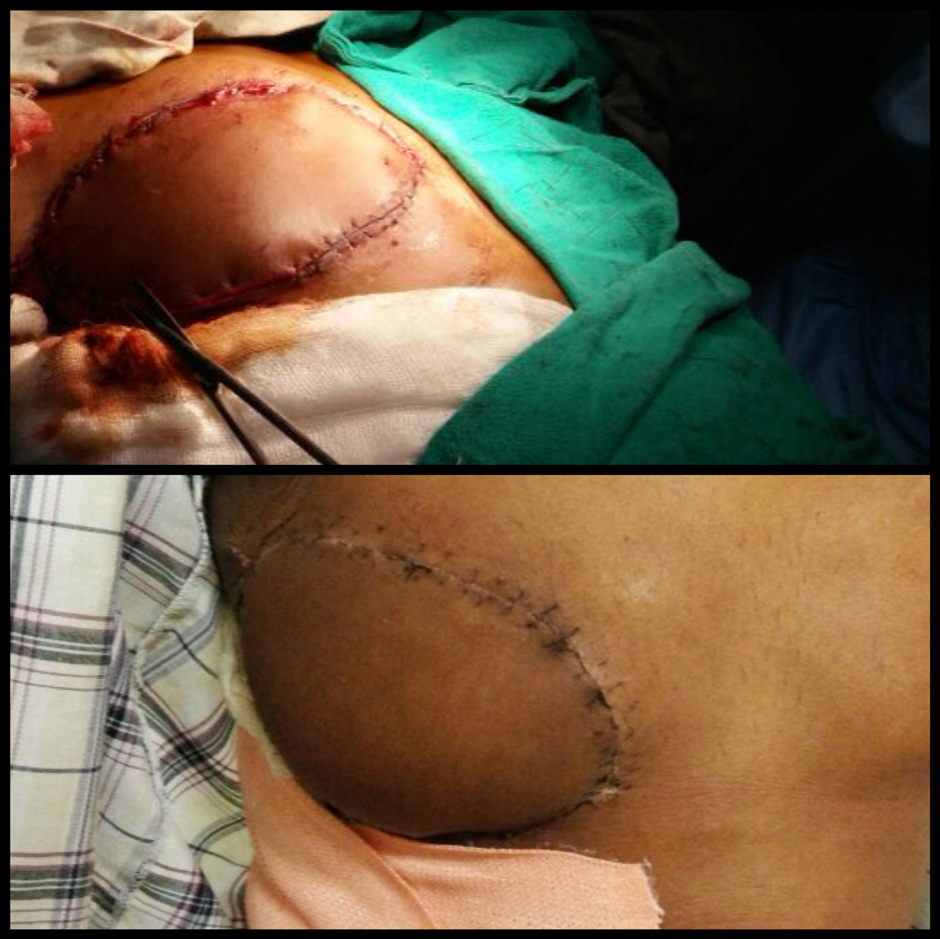
Reconstruction with myocutaneous LD flap after WLE of the tumour. Intra-operative view (top). Post-operative view (bottom).

**Figure 4.**
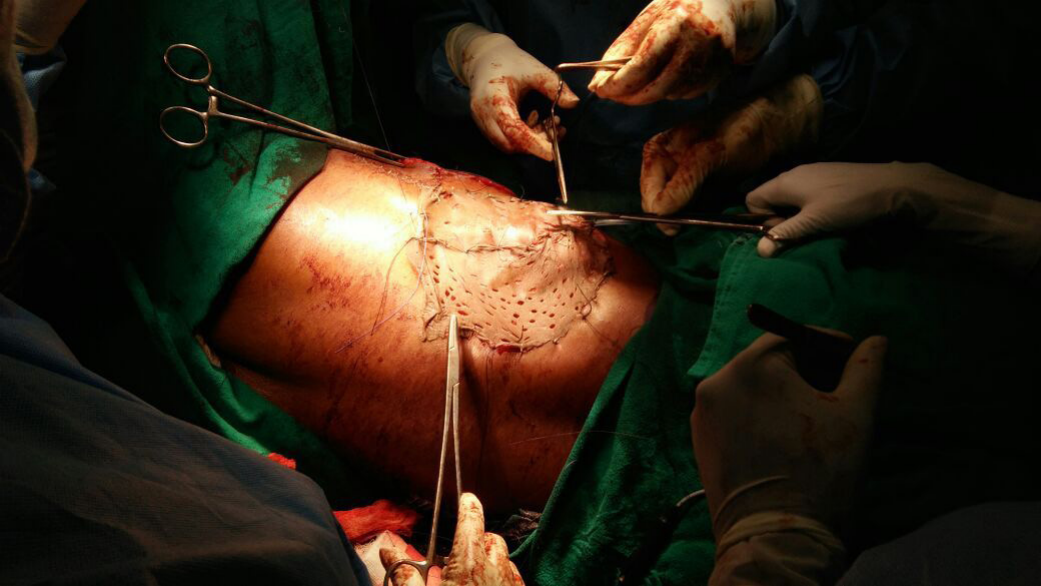
Donor site covered with split skin graft from right thigh.

An elliptical incision was made around the tumour, taking 2.5-cm margin including the previous surgical scar and right nipple-areola complex due to its proximity. The tumour was excised, taking a 2.5-cm circumferential margin, including the surrounding breast tissue and extending up to the pectoralis major fascia. On assessing the tumour grossly, it was felt that it was reaching up to deep margin. Therefore, an intra-operative decision was taken to do a cavitary shave of the deep margin including 1 cm of pectoralis major muscle. The LD flap was raised and placed over the defect (Figures 2 and 3). The donor site was covered with a skin graft from the right thigh (Figure 4).

**Figure 5.**
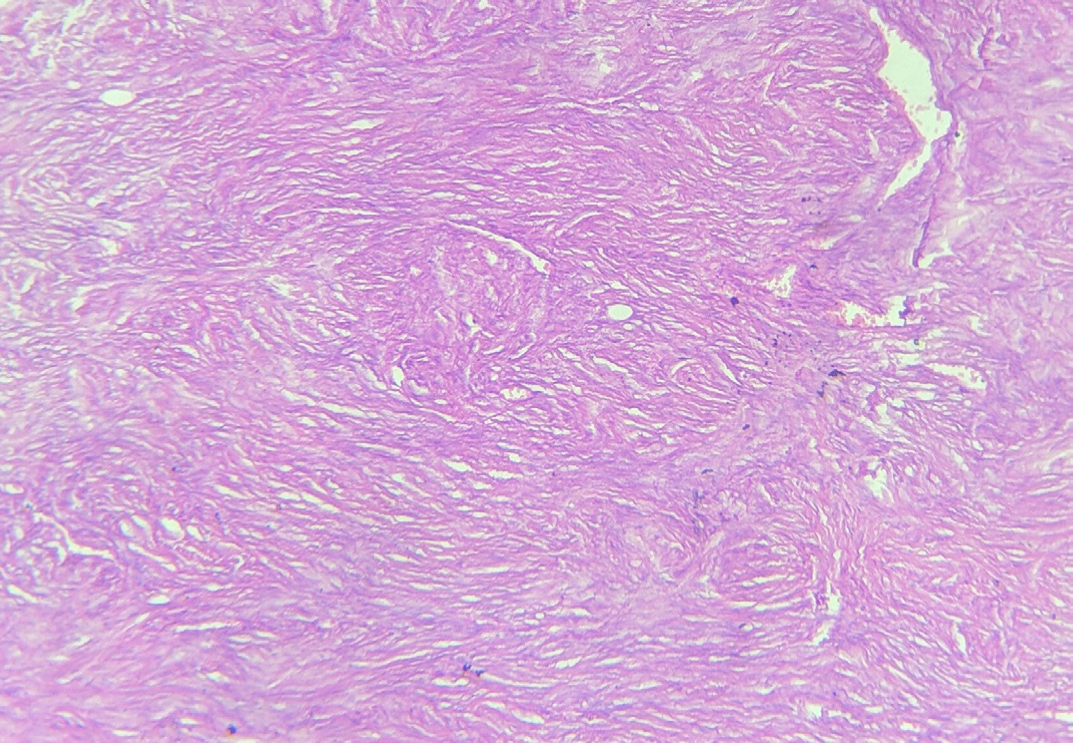
Histopathology of the specimen showed spindle cell tumour with myxoid changes and areas of necrosis and low mitotic count (1–2/hpf)—diagnostic of DFSP.

The wide local specimen measured 12 × 9 × 3 cm with nipple-areola complex at the edge of the skin flap. On serial sectioning, a grey-white tumour measuring 5 × 4 × 3 cm was identified. Histopathology of the tumour showed a spindle cell tumour with myxoid changes and areas of necrosis and low mitotic count (1–2/hpf) (Figure 5). The circumferential margins, skin, nipple and areola were free of the tumour. Tumour cells extended up to deep resected margins of the initial specimen and were 0.1 cm away from it. The Pectoralis major fascia and the shaved part of muscle were free from tumour invasion, achieving a histologically deep free margin of 2 cm. Immunohistochemistry was diagnostic of DFSP-positive for CD34 and vimentin and negative for S-100 and smooth muscle actin. Thus, confirming the diagnosis of recurrent DFSP with myxoid changes.

The post-operative period was uneventful with good functional results. The patient was then referred to the Medical Oncology Department for consideration of imatinib therapy and/or radiotherapy. There were no signs of recurrence at 6 months follow-up.

## Discussion

DFSP is an uncommon, low-grade sarcoma of dermal fibroblast origin with an incidence rate of 4.2–4.5 cases per million persons per year [2]. The median age of presentation is 38.5 years with roughly equal distribution between males and females [4]. The trunk and extremities are considered the most common sites for DFSP; nevertheless, it has been described in various parts of the body including the neck, head, breast and even vulva [3].

DFSP has been reported to arise in areas with a history of prior trauma, including tattoos, vaccination sites, burn scars, surgical scars and radiation treatment. The exact mechanism in which trauma may predispose for development of DFSP is unknown, but it seems intuitive that chronic inflammation and stimulation of the immune system at a local level may trigger the immunopathologic changes that could lead to the malignant transformation of dermal cells [4]. Studies have shown that DFSP demonstrates the chromosomal translocation *t*(17; 22)(*q*22; *q*13) between chromosomes 17 and 22, which is thought to be a key in the tumour’s pathogenesis. The chromosomal translocation leads to the fusion of platelet-derived growth factor (PDGF) beta polypeptide gene and collagen type 1A1 gene, resulting in the overproduction of PDGF and eventually cellular proliferation and tumour formation. This chromosomal translocation is present in over 90% of DFSP and can be detected either by fluorescence *in situ* hybridisation (FISH) on interphase nuclei and/or by multiplex reverse transcription-polymerase chain reaction (RT-PCR) [5]. Other chromosomal translocations like the COL6A3-PDGFD fusion gene have been associated with an apparent predilection for breast [6]. This particular translocation is not detected by conventional RT-PCR used for DFSP and requires RNA sequencing and FISH for detection [6].

The early clinical symptoms of DFSP are non-specific, making diagnosis difficult and leading to a high incidence of misdiagnosis [7]. The most frequent presentation described in adults is a large plaque presenting multiple nodules on its surface [8]. Due to lack of pathognomonic clinical findings, DFSP can be mistaken for a keloid, hypertrophic scar, sebaceous cyst or lipoma and is often referred late for specialised evaluation [4]. In our case, the lack of characteristic clinical appearance of keloid/hypertrophic scar as well as prior history of DFSP with irregular growth raised a suspicion of recurrence. Therefore, a history of prior trauma associated with an ill-defined cutaneous lesion warrants consideration of DFSP in the differential diagnosis [4].

Pathological and immunohistochemical examinations are currently the gold standard for diagnosing DFSP [7]. Microscopically, it demonstrates a uniform population of monomorphic spindle cells arranged in a characteristic storiform pattern over a background of fibrous stroma. These cells may show infiltration into the surrounding subcutaneous fat. The lesions show little nuclear pleomorphism and have low to moderate mitotic activity. They occasionally show myxoid or densely collagenous areas or areas of haemorrhage [3]. Three-dimensional reconstruction of DFSP has shown tumours with highly irregular shapes and frequent finger-like extensions. As a result, incomplete removal and subsequent recurrence are common. The local recurrence rate for DFSP is reported up to 60% [2]. Several histopathological variants of DFSP have been described including pigmented DFSP or Bednar tumour, myxoid, juvenile DFSP or giant cell fibroblastoma, atrophic, sclerosing and myoid, occurring in pure form or admixed with one of the others creating hybrid lesions [9]. Fibrosarcomatous DFSP is also an atypical DFSP subtype, with high rates of recurrence and metastasis [7]. Immunohistochemical studies reveal strong staining with human progenitor cell antigen CD34, but negative for factor XIIIa [3]. In our case, the histopathological assessment revealed findings diagnostic of classical DFSP with myxoid changes (<50%) along with positive immunostaining with CD34 and vimentin. It was diagnosed as a classical DFSP as a diagnosis of myxoid variant requires the presence of greater than 50% of myxoid areas in the tumour [9].

The role of radiological imaging is limited in the management of DFSP. Most patients undergo excision and biopsy of the mass without the need for CT or magnetic resonance imaging (MRI) due to its superficial location [3]. On CT, the tumours appear as superficial well-defined nodular masses involving the skin and subcutaneous fatty tissue with attenuation values approaching that of skeletal muscle and a moderate degree of contrast enhancement. On MRI, typical lesions demonstrate prolonged T1 and T2 relaxation times (hyperintense on T2 and hypointense on T1 weighted images) and signal characteristics, which are considered nonspecific and demonstrated by many other benign and malignant soft tissue condition. Cases of DFSP with atypical locations do not show much radiological difference from that found in the typical common locations such as trunk and extremities [3]. However, imaging assumes an important role in atypical cases or deep-seated lesions for the definition of margins and local invasion as a part of the preoperative analysis. In addition, metastasis, although rare, can be ruled out with imaging. In our case, we performed CT to assess invasion into surrounding structures as well as to rule out metastasis, as this was an atypical case with recurrence.

The treatment of DFSP is primarily surgical. The size and location of the tumour and cosmetic issues will dictate the most appropriate surgical procedure. Modified Mohs surgery and traditional WLE, typically with 2–4-cm margins to investing fascia that are subsequently verified to be clear through the traditional pathologic examination, are all methods to achieve complete histologic assessment [2]. Moh’s surgery offers maximal tissue conservation and better margins as compared to WLE but the local recurrence rates are statistically similar [2]. Mohs micrographic surgery is preferred in regions such as the head and neck regions, where tissue conservation is an important issue [3]. However, the lack of expertise in all centres results in using the technique of WLE with or without plastic reconstruction as the surgery of choice [10].

Margin status is important in the surgical management of DFSP as it determines further treatment and prognosis. Disease-free survival after treatment for DFSP is strongly predicted by tumour depth in the primary setting and margin status in recurrent tumours [11]. However, because of its proclivity for irregular and frequently deep sub-clinical extensions [2], these ‘tricky’ margins pose a challenge to surgical dexterity. In the present case, as it was a recurrent tumour, we opted for a WLE approach to give us as much margin as possible, while preserving the muscular function due to its proximity to pectoralis major. We achieved a maximum possible margin of 2 cm after resection of pectoralis major fascia as well as part of the muscle. Reconstruction was done by using the LD flap, as the flap provides excellent coverage of defects as well as the opportunity to start radiotherapy sooner, because of its better tolerability to radiation compared to grafts, which require time for maturation, delaying the onset of radiotherapy.

Radiation therapy, if not given previously, or imatinib mesylate should be considered if this is not possible, or if additional resection would lead to unacceptable functional or cosmetic outcomes. Imatinib mesylate, a protein tyrosine kinase inhibitor, has been approved by the FDA for the treatment of un-resectable, recurrent and/or metastatic DFSP in adult patients as it inhibits overactivity of PDGF receptor in these tumour cells [2]. Because of the recurrent nature of DFSP, our patient was referred for consideration of chemotherapy with imatinib and radiotherapy as additional resection will lead to a functional loss, in case of recurrence in the future.

In a prospective study of 244 patients [12], the median time to recurrence, local and distal, was 35 months. However, recurrences after long latency have also been reported, including 8 years in the present case. This emphasises the importance of follow-up in DFSP cases, as well as advocating for a long duration of self-surveillance of surgical scars by patients themselves, as it is not always practical to follow every patient [11].

## Conclusion

In conclusion, DFSP is an uncommon, low-grade sarcoma of dermal fibroblast origin with a high local recurrence rate. Diagnosis is established by histology and immunohistochemistry. Treatment of DFSP is primarily surgical—Modified Mohs surgery or traditional WLE. The size and location of the tumour, and cosmetic issues, will dictate the most appropriate surgical procedure. The disease-free period in DFSP is strongly predicted by margin status in recurrent tumours; therefore, every effort should be made to achieve histologically free margins. Recurrences can occur after a long latent period, like 8 years in the present case. The current case emphasises the importance of follow-up in DFSP cases, as well as advocating long duration self-surveillance of surgical scars by patients.

## Conflicts of interest

The authors have no conflicts of interest to declare.

## Financial support/source of funding

The authors have received no funding for this work.

## Authors’ contributions

DKD was responsible for data acquisition and contributed to the design and editing of the manuscript. NW wrote the manuscript and contributed to the design and editing. SP critically reviewed the final draft.
